# Selective Cytotoxic Action and DNA Damage by Calcitriol-Cu(II) Interaction: Putative Mechanism of Cancer Prevention

**DOI:** 10.1371/journal.pone.0076191

**Published:** 2013-09-27

**Authors:** Asim Rizvi, S. Saif Hasan, Imrana Naseem

**Affiliations:** 1 Department of Biochemistry, Faculty of Life Sciences, Aligarh Muslim University, Aligarh, India; 2 Department of Biological Sciences, Hockmeyer Hall of Structural Biology, Purdue University, West Lafayette, Indiana, United States of America; National Institutes of Health, United States of America

## Abstract

**Background:**

Vitamin D is known to play an important role in cancer-prevention. One of the features associated with the onset of malignancy is the elevation of Cu (II) levels. The mode of cancer-prevention mediated by calcitriol, the biologically active form of vitamin D, remain largely unknown.

**Methods:**

Using exogenously added Cu (II) to stimulate a malignancy like condition in a novel cellular system of rabbit calcitriol overloaded lymphocytes, we assessed lipid peroxidation, protein carbonylation, DNA damage and consequent apoptosis. Free radical mediators were identified using free radical scavengers and the role of Cu (II) in the reaction was elucidated using chelators of redox active cellular metal ions.

**Results:**

Lipid peroxidation and protein carbonylation (markers of oxidative stress), consequent DNA fragmentation and apoptosis were observed due to calcitriol-Cu (II) interaction. Hydroxyl radicals, hydrogen peroxide and superoxide anions mediate oxidative stress produced during this interaction. Amongst cellular redox active metals, copper was found to be responsible for this reaction.

**Conclusion:**

This is the first report implicating Cu (II) and calcitriol interaction as the cause of selective cytotoxic action of calcitriol against malignant cells. We show that this interaction leads to the production of oxidative stress due to free radical production and consequent DNA fragmentation, which leads to apoptosis. A putative mechanism is presented to explain this biological effect.

## Introduction

Vitamin D_3_ is obtained from food (fortified dairy products and fish oils) or is synthesized in the skin from 7-dehydrocholesterol by ultraviolet irradiation. In the liver, vitamin D_3_ is hydroxylated at C-25 by vitamin D 25 hydroxylase or cytochrome P450 and forms 25-hydroxyvitamin D_3_ (25(OH)D_3_). 25(OH)D_3_ is then transported to the kidney where in the proximal renal tubule it is hydroxylated at C-1 resulting in the formation of the hormonally active from of vitamin D, 1,25-dihydroxyvitamin D_3_ (1,25(OH)_2_D_3_) or calcitriol which is an essential nutrient that mediates a variety of metabolic processes [1]. Various lines of evidence based on pharmacology and *in vivo* animal model studies show that calcitriol is an anti-cancer agent *in vivo* [2], which acts by initiating apoptosis and inhibiting the growth of several malignant cell lines [3]. Epidemiological studies show that less exposure to sunlight and consequent lower levels of calcitriol increase the risk of cancer and mediate the progression of several cancers, which include cancers of breast, ovary, colon, oesophagus, rectum, pancreas and blood [4,5]. The wide variety of malignant cells that respond to fluctuating levels of calcitriol indicates the widespread role of calcitriol in mediating anti-cancer effects. However, the exact mechanism by which anti-cancer effects of calcitriol are effected remains unknown.

One of the characteristic features of malignancy is the elevation of copper levels [6–8]. Copper is an important metal ion found to be associated with chromatin DNA, particularly with guanine [9]. It is known that in malignant cells, copper concentrations may be elevated two-to-five times, as compared to those reported in normal cells [10]. Although the precise reason for elevation in copper levels during malignancy remains unclear, increased angiogenesis is thought to be a possible reason [11].

In this study, we propose a physiological link between calcitriol and copper in malignant cells. We describe a putative, Cu (II)-induced, free radical-mediated pathway, which explains the selective cytotoxic action of calcitriol against malignant cells. To illustrate calcitriol activity, we have used a cellular system of calcitriol overloaded lymphocytes (COLs). We demonstrate that COLs, when exposed to Cu (II) ions to simulate the environment of a malignant cell, undergo oxidative damage and DNA breakage, which eventually leads to cell death. Since calcitriol, an essentially hydrophobic species, affects DNA, a highly polar molecule, we present a hypothesis to explain the interaction between calcitriol and DNA. Based on mutagenesis, biochemical and structure-related data [12–18], we discuss the possibility that the Vitamin D Receptor (VDR) serves as an “adaptor protein” that mediates this process. We utilize the recently elucidated structure of VDR and its binding partner RXR (retinoic X receptor) [19] to explain our hypothesis. To the best of our knowledge, the present research is the first report which shows that Cu (II) plays a role in calcitriol-mediated cell death.

## Material and Methods

### Chemicals

All chemicals and enzymes used were obtained from Sigma Aldrich (USA). All solutions were prepared the same day and used immediately.

### Ethical Statement for Animal Experimentation

Animal experimentations were permitted by Ministry of Environment and Forests, Government of India under registration no 714/02/a/CPCSEA issued by Committee for the Purpose of Control and Supervision of Experiments on Animals (CPCSEA) dated 16th November, 2002 and approved by the institutional ethical committee of Department of Biochemistry, Aligarh Muslim University, Aligarh, India (Order no: DNo1).

### Preparation of Calcitriol Overloaded Lymphocytes (COLs)

Fifteen male albino rabbits weighing 1 + 0.1 kg were purchased and randomly divided into three groups of five rabbits each. The rabbits were housed in individual steel cages, and maintained on standard rabbit chow and water, provided *ad libitum*, with 12 hr light and dark cycles at 25°C. The animals were acclimatized for a month before beginning the experiment. Animals in group one received 200 ng/g body weight of cholecalciferol dissolved in 1 ml of ethanol, intraperitonially every day for a period of two weeks. Animals in group two received intraperitonial injections of 1 ml ethanol (vehicle control) and animals in group three served as controls. After two weeks the animals were sacrificed and blood was collected in heparinised tubes and diluted in ion-free phosphate buffered saline (pH 7.0). Lymphocytes were isolated using Histopaque 1077 and the cells were suspended in RPMI 1640. Freshly isolated lymphocytes were used for all experiments.

### Determination of Intracellular Calcitriol Levels in COLs

Isolated lymphocytes were lysed and the cell lysate was further used to determine levels of calcitriol, using an USCN Life Sciences Inc. (Huston, Texas, U.S.A.) kit according to the manufacturer’s instructions.

### Treatment of Lymphocytes

Lymphocytes were suspended in a total volume of 3 ml PBS and incubated in the presence of Cu (II) (25 µM) for 2 hours. The reaction was also performed in the presence of specific metal ion chelators. Desferoxamine (50 µM) was used to chelate Fe (II) ions, histidine (50 µM) was used to chelate Zn (II) and bathocuprione and neucuprione (50 µM each) were used to chelate extracellular and intracellular Cu (II) ions. Free radical scavengers (catalase 20 µg/ml, superoxide dismutase (SOD) 20 µg/ml, and thiourea 0.1 mM) were used in a separate set of reactions; to implicate the role of ROS in Cu (II) mediated oxidative stress. Equal numbers of lymphocytes (1 x 10^8^ + 10^3^) were used in all experiments.

### Cu (II) induced lipid peroxidation in COLs

Thiobarbituric Acid reactive Substances (TBARS) were estimated in lymphocytes by the method of Ramanathan et al. [20], with minor modifications. To 1.5 ml reaction mixture, 0.5 ml of 10% TCA (trichloroacetic acid) and 0.5 ml of 0.6 M TBA (2-thiobarbituric acid) were added and the mixture was incubated in a boiling water bath for 20 minutes. The absorbance was read at 532 nm and converted to nano-moles of TBA reactive substances using the molar extinction coefficient.

### Cu (II) induced protein carbonylation in COLs

Treated lymphocytes were lysed and the amount of carbonyl groups formed was determined [21]. One ml reaction mixture after treatment was blended with 0.5 ml of 10 mM 2,4-dinitrophenylhydrazine in 2.5 M HCl. The mixture was left for 1 hr at room temperature and 0.5 ml of 20% trichloroacetic acid was added to the tube. The tube was left in an ice bucket for 10 mins followed by centrifugation at 12,000 x g for 15 min. The supernatant was discarded and the protein pellet was washed with ethanol/ethyl acetate (1:1 v/v) and dissolved in 2 ml of 6 M guanidine (pH 2.3) and vortexed. The carbonyl content was calculated using the molar absorption coefficient of 22,000 M^-1^ cm^-1^

### Comet, Assay (Single Cell Alkaline Gel Electrophoresis) of COLs

Comet assay was performed using the method employed by Singh et al. [22], with minor modifications. Fully frosted slides were precoated with 1.0% normal melting agarose. Lymphocytes were mixed with 90 µl of 1.0% low melting point agarose to form a cell suspension and pipetted over the first layer and covered with a cover slip. The slides were placed on ice packs for 10 minutes to solidify the agarose. The coverslips were gently removed and a third layer of 0.5% low melting point agarose was pipetted. The slides were covered with cover slips and were placed on ice packs to solidify. The cover slips were then removed and the slides were immersed in ice cold lysis buffer for an hour. After lysis, the DNA was allowed to unwind in alkaline electrophoretic solution (300 mM NaOH, 1 mM EDTA, pH<13). Electrophoresis was performed at 4°C, in a field strength of 0.7 V/cm and 300 mA current. The slides were neutralised with ice cold 400 mM Tris (pH 7.5), and stained with 75 µl ethidium bromide (20mg/ml) and covered with a cover slip. Slides were scored using an image analysis system (Komet 5.5, Kinetic Imaging, Liverpool, UK) attached to an Olympus (CX41) fluorescent microscope (Olympus Optical Co, Tokyo, Japan) and a COHU 4910 integrated CC camera (equipped with 510-560 nm excitation and 590 nm barrier filters) (COHU, San Diego, CA, USA). Images of 25 cells were analysed from each triplicate slide. Tail length (migration of DNA from nucleus in µmeters) was the parameter used to asses DNA damage.

### Cu (II) induced apoptosis in COLs

Treated lymphocytes were smeared on a clean, ethanol sterilized glass slide, and were stained with Leishman’s stain. Apoptotic cells were visually scored in three randomly selected visual fields according to criteria of Hasan et al. [23] using a Nikon binocular compound microscope.

### Statistical analysis

Values are expressed as mean + SEM from three independent experiments. Data was analysed by Tukey’s test after one way analysis of variance (ANOVA) using GraphPad Prism 5.01 (California, USA). Differences were considered statistically significant at P < 0.05

## Results

### Cellular Model to study ex-vivo interaction of calcitriol and copper

**Figure 1:**
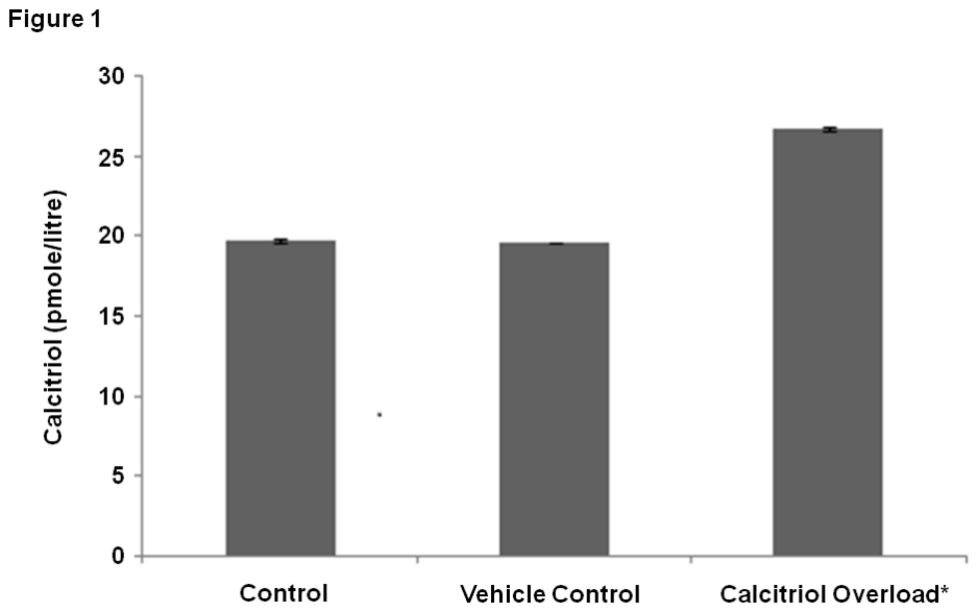
Calcitriol Overloaded Lymphocytes (COLs) Levels of calcitriol in calcitriol overloaded lymphocyte (COLs) lysates and control lymphocytes. Values expressed as mean + SEM (n=3) * P <0.001 as compared to control and vehicle control.

As mentioned above calcitriol is known to mediate anti-cancer effects. It has been reported that Cu (II) is also significantly elevated in malignant cells. To study the effects of calcitriol-Cu (II) interaction, we have developed a model system based on lymphocytes. Calcitriol overloading of lymphocytes was achieved via intraperitoneal injection of the calcitriol-precursor cholecalciferol, which underwent conversion into calcitriol, within the animal system. Following cholecalciferol injection, it was observed that calcitriol levels in isolated lymphocytes were elevated by 25.52% (Figure 1). Cu (II) levels were increased by *in vitro* supplementation of isolated, COLs with Cu (II). Assays for lipid peroxidation, protein carbonylation and DNA damage as a consequence of calcitriol-Cu (II) interaction were performed on the isolated lymphocyte system.

### Cu (II) induced lipid peroxidation in COLs

**Figure 2:**
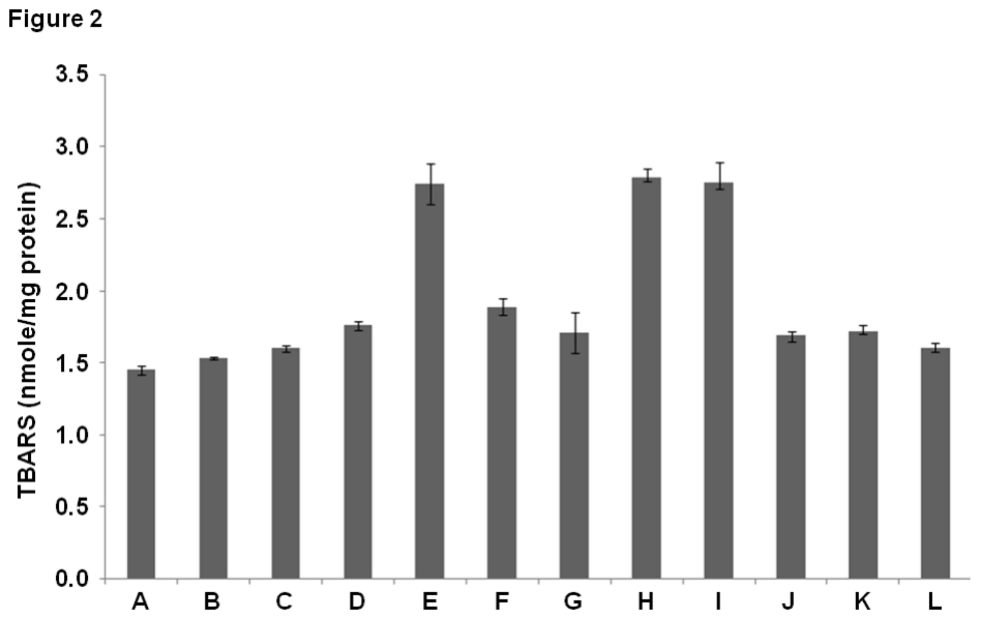
Assay of lipid peroxidation (A) Control (B) Vehicle control (C) Cu (II) (25µM) [added to non calcitriol loaded, control lymphocytes] (D) Calcitriol overload (E) Calcitriol overload + Cu (II) (25µM) (F) Calcitriol overload + Cu (II) (25µM) + Bathocuprione (50µM) (membrane impermeable Cu(II) chelator) (G) Calcitriol overload + Cu (II) (25µM) + Neucuprione (50µM) (membrane permeable Cu(II) chelator) (H) Calcitriol overload + Cu (II) (25µM) + Desferoxamine (50µM) (Fe(II) chelator) (I) Calcitriol overload + Cu (II) (25µM) + Histidine (50µM) (Zn(II) chelator) (J) Calcitriol overload + Cu (II) (25µM) + SOD (20µg/ml) (K) Calcitriol overload + Cu (II) (25µM) + Catalase (20µg/ml) (L) Calcitriol overload + Cu (II) (25µM) + Thiourea (0.1mM). Values expressed as mean + SEM (n=3). P < 0.05 when compared to control and COLs with Cu (II) (25µM). All incubations were carried out for 2 hours at 37º C.

Calcitriol has been shown to cause oxidative stress. One of the consequences of oxidative stress is the peroxidation of cellular lipids [20]. Hence, lipid peroxidation was employed as a marker to assess oxidative stress as a function of calcitriol levels. Elevation in calcitriol levels did not lead to significant lipid peroxidation in COLs (Figure 2). However, exposure of COLs to Cu (II) ions led to a marked increase in lipid peroxidation (Figure 2). To determine if lipid peroxidation is a Cu (II)-specific effect, or if other redox-active divalent metal ions, such as Fe (II) and Zn (II) mediate lipid peroxidation in the presence of calcitriol, chelators of Fe (II) and Zn (II) ions (desferoxamine and histidine, respectively) were added to isolated COLs. It was observed that removal of Fe (II) and Zn (II) did not cause significant inhibition of lipid peroxidation (Figure 2). As a control, chelators of Cu (II) were introduced to test the specific role of Cu (II) in causing oxidative stress in COLs. It was observed that significant inhibition of lipid peroxidation was achieved upon removal of Cu (II) (Figure 2). To identify the location of the Cu (II) pool (intra-cellular versus extracellular) that mediates lipid peroxidation, the effect of Cu (II) chelators with different membrane permeabilities was tested on lipid peroxidation in isolated COLs. It is significant to note that bathocuprione, which is a membrane-impermeable Cu (II) chelator, did not inhibit lipid peroxidation as effectively as neucuprione, a membrane-permeable copper chelator (Figure 2). It is inferred that the intracellular pool of Cu (II) mediates lipid peroxidation through interactions with calcitriol. It was observed that exogenously added Cu (II) in control (unloaded lymphocytes) did not induce significant lipid peroxidation (Figure 2). As peroxidation is caused by the activity of agents of oxidative damage, free radical scavengers (SOD, catalase and thiourea) were used to determine the identity of the free radicals that are involved in Cu (II)-calcitriol induced oxidation events. Effective inhibition of Cu (II)-induced lipid peroxidation in isolated COLs was observed with SOD, catalase and thiourea (Figure 2). It is inferred that the superoxide anion, hydrogen peroxide and hydroxyl radicals, scavenged respectively by SOD, catalase and thiourea, mediate Cu (II)-induced lipid peroxidation.

### Cu (II) induced protein carbonylation in COLs

**Figure 3:**
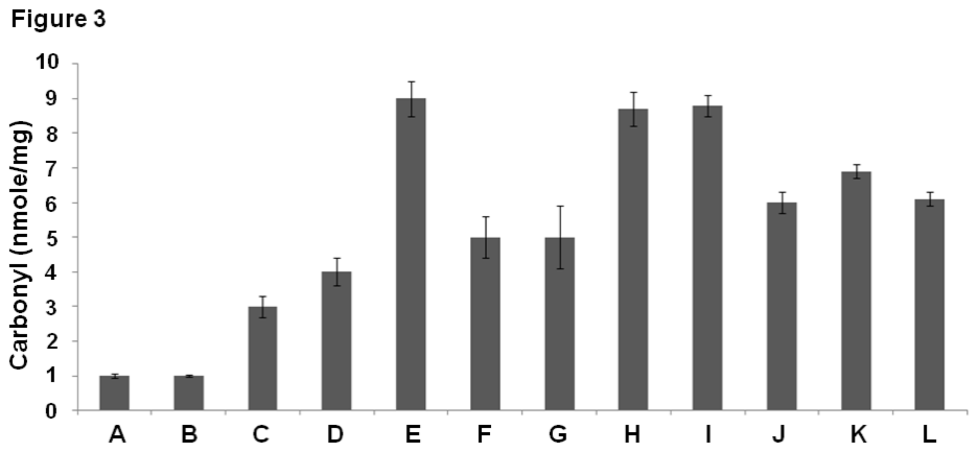
Assay of protein carbonylation (A) Control (B) Vehicle control (C) Cu (II) (25µM) [added to non calcitriol loaded, control lymphocytes] (D) Calcitriol overload (E) Calcitriol overload + Cu (II) (25µM) (F) Calcitriol overload + Cu (II) (25µM) + Bathocuprione (50µM) (membrane impermeable Cu(II) chelator) (G) Calcitriol overload + Cu (II) (25µM) + Neucuprione (50µM) (membrane permeable Cu(II) chelator) (H) Calcitriol overload + Cu (II) (25µM) + Desferoxamine (50µM) (Fe(II) chelator) (I) Calcitriol overload + Cu (II) (25µM) + Histidine (50µM) (Zn(II) chelator) (J) Calcitriol overload + Cu (II) (25µM) + SOD (20µg/ml) (K) Calcitriol overload + Cu (II) (25µM) + Catalase (20µg/ml) (L) Calcitriol overload + Cu (II) (25µM) + Thiourea (0.1mM). Values expressed as mean + SEM (n=3). P < 0.05 when compared to control and COLs with Cu (II) (25µM). All incubations were carried out for 2 hours at 37º C.

Protein carbonylation is a consequence of oxidative stress and the interaction of free radicals with amino acid side chains. Thus protein carbonylation serves as a marker for the oxidative stress encountered by a biological system. Exposing COLs to Cu (II) ions resulted in a marked increase in total protein carbonylation. Chelating intracellular Zn (II) and Fe (II) did not affect protein carbonylation whereas chelating Cu (II) from the system resulted in a marked decrease in the total carbonyl content. The presence of free radical scavengers (SOD, catalase and thiourea) in the system resulted in a marked decrease in the total protein carbonyl content (Figure 3).

### Cu (II) induced cellular DNA damage in COLs

**Figure 4:**
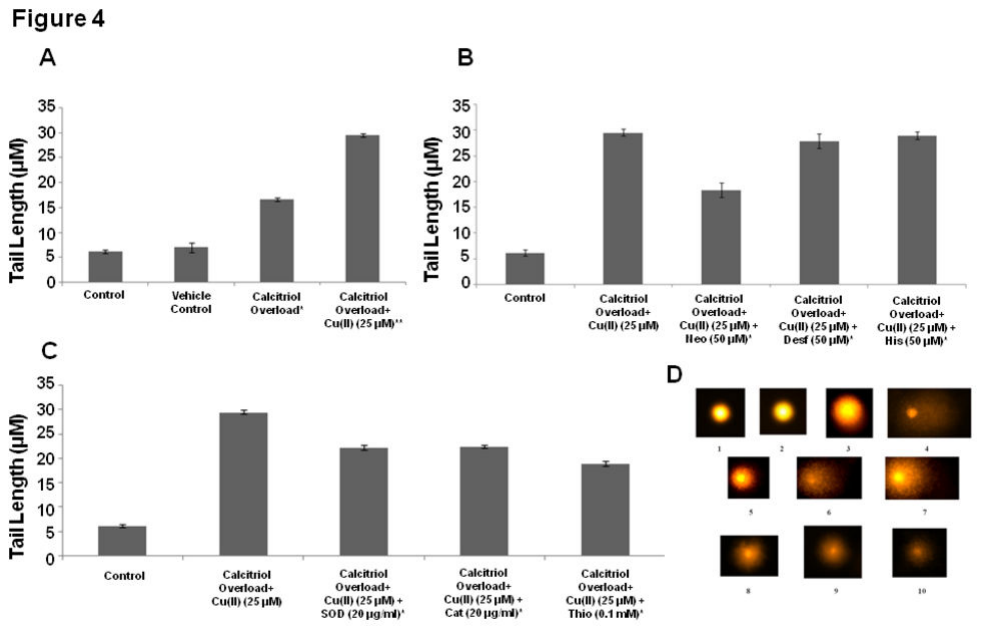
Assay of DNA damage in COLs (A) DNA damage in control lymphocytes, vehicle control lymphocytes, COLs and COLs with Cu (II) (25µM). Values expressed as mean + SEM (n=3) ** P < 0.001as compared to control and vehicle control, P < 0.001 when * and ** are compared). (B) Removal of Cu (II) and DNA damage. COLs with Cu (II) (25µM) + neucuprione (Neo) (50µM) (membrane permeable Cu(II) chelator), desferroxamine (Desf) (50µM) (Fe(II) chelator), histidine (His) (50µM) (Zn(II) chelator) Values expressed as mean + SEM (n=3).*P < 0.001 when compared to control and COLs with Cu (II) (25µM). (C) COLs with Cu (II) (25µM) were incubated along with superoxide dismutase (SOD) (20µg/ml), catalase (Cat) (20µg/ml), and thiourea (Thio) (0.1mM) Values expressed as mean + SEM (n=3). (D) Representative images of Comet Assay (1) Control (2) Vehicle Control (3) COLs (4) COLs + Cu (II) (25µM) (5) COLs + Cu (II) (25µM) + Neo (50µM) (6) COLs+ Cu (II) (25µM) + Desf (50µM) (7) COLs+ Cu (II) (25µM) + His (50µM) (8) COLs+ Cu (II) (25µM) + SOD (20µg/ml) (9) COLs+ Cu (II) (25µM) + Cat (20µg/ml) (10) COLs+ Cu (II) (25µM) + Thio (0.1mM). *P < 0.001 when compared to control and COLs with Cu (II) (25µM). All incubations were carried out for 2 hours at 37º C.

Generation of free radicals causes DNA damage, which leads to apoptosis [24]. One of the characteristics of apoptosis is the breakage of DNA into fragments, which is detected by a comet assay. It was observed that calcitriol overload in lymphocytes resulted in DNA damage, which was significantly enhanced upon incubation of COLs with Cu (II) (Figure 4A, 4D). To further determine the specificity of Cu (II) in mediating DNA damage, the membrane permeable Cu (II)-chelator neucuprione was utilized. Incubation of isolated COLs with neucuprione significantly inhibited DNA damage, implicating intracellular Cu (II) as a participant in calcitriol-mediated DNA damage (Figure 4B, 4D). The involvement of other divalent cations in calcitriol-mediated DNA damage in lymphocytes was tested with desferoxamine and histidine, which did not inhibit DNA damage, ruling out the involvement of Fe (II) and Zn (II) in the process (Figure 4B, 4D). Inhibition of DNA damage by SOD, catalase and thiourea implies that DNA damage is caused by the superoxide anion, hydrogen peroxide and hydroxyl radicals (Figure 4C, 4D).

### Cu (II) induced apoptosis in COLs

**Figure 5:**
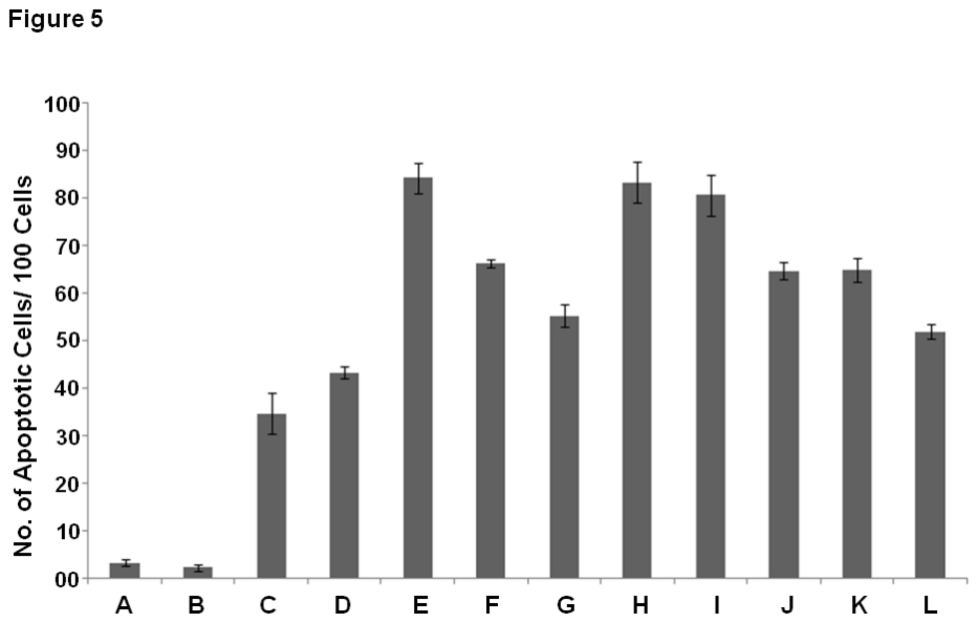
Apoptosis: Number of Apoptotic cells (cells with nuclear fragmentation and/or with distinct apoptotic bodies) per hundred cells. (A) Control (B) Vehicle control (C) Cu (II) (25µM) [added to non calcitriol loaded, control lymphocytes] (D) Calcitriol overload (E) Calcitriol overload + Cu (II) (25µM) (F) Calcitriol overload + Cu (II) (25µM) + Bathocuprione (50µM) (membrane impermeable Cu(II) chelator) (G) Calcitriol overload + Cu (II) (25µM) + Neucuprione (50µM) (membrane permeable Cu(II) chelator) (H) Calcitriol overload + Cu (II) (25µM) + Desferoxamine (50µM) (Fe(II) chelator) (I) Calcitriol overload + Cu (II) (25µM) + Histidine (50µM) (Zn(II) chelator) (J) Calcitriol overload + Cu (II) (25µM) + SOD (20µg/ml) (K) Calcitriol overload + Cu (II) (25µM) + Catalase (20µg/ml) (L) Calcitriol overload + Cu (II) (25µM) + Thiourea (0.1mM). Values expressed as mean + SEM (n=3). P < 0.05 when compared to control and COLs with Cu (II) (25µM). All incubations were carried out for 2 hours at 37º C.

Apoptosis is characterised by morphological changes within the cell. Nuclei of apoptotic cells are characterised by formation of distinct apoptotic bodies and nuclear fragmentation. Exposure of COLs to Cu (II) ions resulted in a marked increase in the number of apoptotic cells. Removal of copper from the system decreased the total number of apoptotic cells, whereas chelation of other redox active divalent metal ions (Zn (II) and Cu (II)) did not significantly affect apoptosis. Free radical scavengers successfully decreased the number of apoptotic cells thereby clearly implicating the role of reactive oxygen species in Cu (II) induced apoptosis of COLs (Figure 5).

## Discussion

In this study, we provide preliminary evidence that calcitriol-Cu (II) interaction is significant in causing DNA damage, which may lead to apoptosis. Since it has been demonstrated that Cu (II) is present bound to genomic DNA [9] and it is elevated in malignant cells [6–8], Cu (II) was tested as candidate metal to activate calcitriol chemistry.

The studies described here test the role of calcitriol-Cu (II) interaction on lipid peroxidation, protein carbonylation and DNA damage, three indicators of oxidative stress and consequent apoptosis. For pro-oxidant activity, generation of reactive oxygen species, from calcitriol is required. Several pro-apoptotic drugs [25,26], plant derived molecules [24,27], and vitamin C [28], act to damage DNA by metal ion mediated Fenton chemistry.

**Figure 6:**
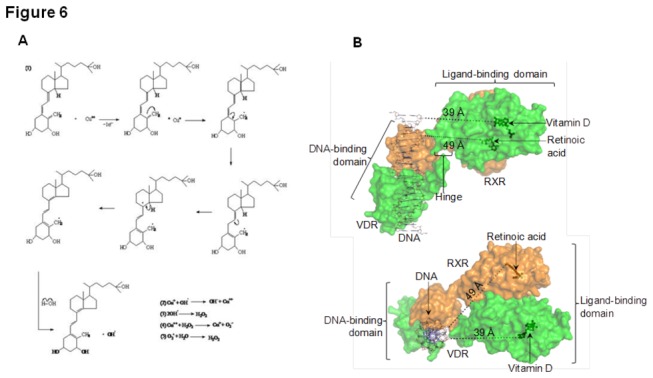
A putative mechanism for calcitriol-Cu (II) interaction. (A) Production of hydroxl radicals, superoxide anions and hydrogen peroxide lead to DNA damage and consequent cell death. (B) Hetero-dimer of VDR (green) and RXR (orange) showing separation between the calcitriol binding site and bound DNA. Upper panel: View normal to axis of bound DNA. Lower panel: View along axis of bound DNA. Calcitriol (dark green sticks and spheres) bound to VDR, constitutes the free radical production site, is separated from the DNA molecule by ~39 Å. For reference, retinoic acid bound to RXR is also shown. The double-stranded DNA molecule is shown as thin black/blue sticks. Distance measurements and figure preparation were performed in PyMol (www.pymol.org) using coordinates of the VDR/RXR hetero-dimer [Orlov et al. EMBO J. 2012] kindly provided by B. Klaholz.

We propose a putative mechanism (Figure 6 A) by which calcitriol may interact with DNA bound Cu (II) to exert its pro-oxidant effects. The methylene group of calcitriol accepts an electron from Cu (II) and by a series of double bond rearrangements and autolysis of water forms hydroxyl radicals. These hydroxy radicals may either combine with each other and lead to the production of hydrogen peroxide or may alternatively interact with Cu (I) and lead to the production of Cu (II), thereby establishing a redox cycle.

Hydrogen peroxide once formed, may further react with Cu (II) leading to its homolytic/hetrolytic breakdown, that forms yet more hydroxyl radicals, and Cu (II) is reduced to Cu (I). Alternatively superoxide anion could react with water and form yet more hydrogen peroxide.

Since intracellular copper is bound to DNA, and calcitriol is known to enter the nucleus, the production of free radicals and hydrogen peroxide by the proposed mechanism, would in all probability, take place in the immediate vicinity of DNA leading to DNA damage. Hydroxyl radicals seem to play a major role in generation of oxidative stress, since thiourea, a potent scavenger of hydroxyl radicals can prevent both lipid peroxidation and DNA damage significantly. It is also significant to note that the membrane permeable Cu (II) chelator, neucuprione also inhibits lipid peroxidation, protein carbonylation, DNA damage and apoptosis, possibly by chelating and thereby, making the DNA bound copper unavailable for reacting with calcitriol.

To rule out the involvement of the other major redox-active metals present in biological systems, Fe (II) and Zn (II) were tested. Experiments with chelators of Fe (II) and Zn (II) did not cause inhibition of calcitriol-mediated lipid peroxidation, protein carbonylation, DNA damage and apoptosis in COLs. Therefore, it is concluded that Cu (II) interacts specifically with calcitriol to mediate DNA damage which leads to apoptosis. Calcitriol is inferred to play a role in the activation of apoptosis in a Cu (II) rich environment, such as that observed in malignant cells, which may act as a defence mechanism to control malignant cell growth.

Results described in the present study provide insights into calcitriol-Cu (II)-DNA interactions. In this regard, it is significant to note that calcitriol, a hydrophobic molecule, interacts with Cu (II) and DNA, which are both highly polar. As a lipid derivative, calcitriol partitions in lipid bilayers. In case of a direct interaction between calcitriol and the polar species, it is imperative that an adaptor molecule brings calcitriol in the vicinity of DNA and DNA bound Cu (II). One such molecule is the vitamin D receptor (VDR). It is well documented that VDR localises in the nucleus [29], and binds selectively to both calcitriol and DNA. For efficient oxidative damage spatial proximity between calcitriol and DNA (along with bound Cu (II) ions) is necessary. Secondly VDR knockout mice have been shown to be extremely sensitive to chemical carcinogens. Within 60 days of exposure, 88% of VDR null mice exposed to the chemical carcinogen DMBA developed cancer [30]. VDR null mice have been shown to be very sensitive to chemically induced lukemia and experimental VDR ablation causes increased skin and mammary gland tumuroginesis [30] indicating a clear role of the VDR in controlling the process of carcinogenesis. It has been proven that VDR expression positively correlates with tumour suppression [30]. A model of the full length VDR structure, in combination with the binding partner retinoic acid receptor (RXR) has been reported through cryo-electron microscopic (cryo-EM) reconstruction recently [12]. It is of significance to note that the VDR hinge region has been shown to be flexible, and this flexibility is crucial to the function of the full-length polypeptide [13–16]. Calcitriol molecule is found to be separated from the bound DNA molecule by a distance of approximately 39 Å in the VDR structure (Figure 6B). We hypothesise that the VDR molecule may undergo a conformational change along the hinge, Figure 6B (upper panel), which brings the bound DNA close to calcitriol [18].

The fact that calcitriol is a proxidant in malignant cells has been demonstrated by Koren et al. [31] who showed that in the MCF-7 cell line, glutathione levels increase upon exposure to calcitriol, which is an indicator of increased ROS generation. Narvaez and Welsh [32] have shown that calcitriol induceded apoptosis of malignant cells is dependent on free radical generation. The commitment of malignant cells to calcitriol-mediated apoptosis was shown to be caspase-independent, indicating that calcitriol-mediated apoptosis of malignant cells is executed by non-classical pathways. Our non enzymatic, copper induced, ROS mediated mechanism leading to irreparable DNA fragmentation may be one of the several factors contributing to apoptotic death of malignant cells upon exposure to calcitriol.
